# Rifampicin-resistant tuberculosis in Fujian Province, Southeast China: a retrospective analysis of drug resistance screening and treatment outcomes, 2019–2024

**DOI:** 10.3389/fpubh.2025.1611459

**Published:** 2025-07-16

**Authors:** Yinfa Zhou, Zhisong Dai, Shufang Lin, Daiquan Chen, Jian Lin, Kun Chen, Yongming Lin, Yanqin Deng

**Affiliations:** ^1^Fujian Center for Disease Control and Prevention, Fuzhou, China; ^2^Fujian Provincial Key Laboratory of Zoonosis Research, Fuzhou, China

**Keywords:** tuberculosis, rifampicin-resistant, treatment outcome, epidemiology, regression analysis

## Abstract

**Backgrounds:**

Rifampicin-resistant tuberculosis (RR-TB) remains a major challenge to global TB control efforts. In Fujian Province, Southeast China, where RR-TB prevalence has been notably high, understanding epidemiological trends and treatment outcomes is crucial for optimizing interventions. This study aimed to analyze RR-TB characteristics, resistance patterns, and treatment outcomes to inform evidence-based control strategies.

**Methods:**

An observational study was conducted utilizing data from China’s National Tuberculosis Information Management System, focusing on bacteriologically confirmed tuberculosis cases reported in Fujian Province during 2019–2024. Epidemiological characteristics, drug resistance and outcomes of RR-TB were described as frequency (n) and percentage (%). Risk factors for unsuccessful outcomes were assessed using univariate and multivariate logistic regression.

**Results:**

A total of 1,368 RR-TB patients were detected, with an overall resistance rate of 3.7%. The RR rate showed a steady decline year by year (*χ^2^* = 76.214, *p* < 0.001), mainly due to the decrease in new TB cases (*χ^2^* = 60.966, *p* < 0.001). RR-TB patients exhibited higher co-resistance to isoniazid (71.9% vs. 6.3%, *p* < 0.001) and ofloxacin (29.8% vs. 1.8%, *p* < 0.001) compared to rifampicin-sensitive TB. Of 1,056 RR-TB patients initiated on treatment, 720 had outcome data, revealing a low success rate (58.6%) due to high loss to follow-up (31.1%) and mortality (9.3%). Multivariate analysis identified male sex (*AOR* = 1.67, *95%* CI: 1.11–2.52, *p* = 0.014), age ≥45 years (*AOR* = 2.27, *95% CI:* 1.58–3.26, *p* < 0.001), high-risk group status (*AOR* = 1.42, *95% CI*: 1.04–1.94, *p* = 0.026), and occupation as farmer/worker (*AOR* = 2.17, *95% CI*: 1.10–4.26, *p* = 0.025) as independent risk factors of unsuccessful treatment.

**Conclusion:**

Fujian Province has demonstrated a steady decline in rifampicin resistance rates, primarily driven by reductions in new TB cases. However, treatment inclusion rate and success rate remains suboptimal, highlighting the need for targeted interventions—including enhanced adherence support, intensive follow-up, and adverse event management—particularly for high-risk groups such as older males and manual laborers. These findings can guide tailored strategies to further reduce RR-TB burden in similar settings.

## Introduction

Tuberculosis (TB), a chronic respiratory infection caused by *Mycobacterium tuberculosis,* continues to be a major public health challenge despite advances in diagnostics and therapeutics (1). One of the most urgent and obstacle to global TB control is the spreading of rifampicin-resistant TB (RR-TB) (2). Previous studies have demonstrated that rifampicin resistance in *Mycobacterium tuberculosis* is predominantly mediated by mutations in the rpoB gene, with additional contributions from efflux pump gene mutations in certain strains. Socioeconomic factors, particularly irregular treatment regimens, exacerbate resistance dynamics by creating selective environments that favor the proliferation of resistant strains over susceptible populations. Furthermore, the transmission of these drug-resistant strains perpetuates the spread of resistance, compounding the public health challenge (3–5). RR-TB encompasses diverse resistance patterns, including mono-resistance, multidrug resistance, and polydrug resistance to rifampicin—a cornerstone of first-line TB treatment. The development of rifampicin resistance complicates clinical management, leading to more toxic and prolonged treatment regimens, extended infectiousness, and significantly higher healthcare costs (6, 7). Thus, to meet the ambitious goal of ending TB by 2035, early detection and effective treatment of RR-TB should be a crucial component.

Globally, the annual incidence of MDR/RR-TB remained relatively stable from 2020 to 2023, with an estimated 400,000 cases (95% UI: 360,000–440,000) and approximately 150,000 deaths (95% UI: 94,000–210,000) reported in 2023. The resistance rates demonstrated significant variation between new TB cases (3.2%; 95% UI: 2.5–3.8%) and previously treated cases (16.0%; 95% UI: 9.0–24%) (1). China represents one of the high-burden countries for RR-TB, with an estimated 29,000 new RR-TB cases reported in 2023, constituting 7.3% of global cases and ranking fourth worldwide in terms of disease burden (1). Surveillance data from Fujian Province, located in southeastern China, demonstrated significantly elevated RR-TB prevalence rates during the 2016–2019 period, with resistance detected in 4.4% of new TB cases and 16.8% of previously treated cases (8). Compared with national/global averages, Fujian’s RR-TB rates in previous monitoring was relatively high. In response to this public health challenge, Fujian Province has implemented comprehensive measures to control RR-TB prevalence, including the enhancement of detection capabilities to achieve a drug resistance screening rate exceeding 90% for bacteriologically confirmed TB cases. Building upon these efforts, our study conducts a analysis of the epidemiological characteristics and treatment outcomes of RR-TB in southeastern China from 2019 to 2024. The study aims to understand the prevalence and treatment outcome of rifampicin resistance in Fujian Province, and to identify modifiable risk factors for unsuccessful treatment outcome. The finding will provide valuable insights and evidence-based recommendations for the development of more precise and effective RR-TB prevention and control strategies in the future.

## Materials and methods

### Data sources

This was an observational study based on data collected directly from China National TB Information Management System (TBIMS). The research population consisted of bacteriologically confirmed TB patients notified in Fujian Province, Southeast China, during 2019–2024. The basic data about the patients and details were obtained from the patient’s medical records in the system.

Fujian Province, a southeastern coastal province of China, has a diverse distribution of coastal areas, encompassing 9 cities in the 3 regions, among which Fuzhou, Putian and Ningde are the eastern region, Xiamen, Zhangzhou and Quanzhou are the southern region, and Longyan, Sanming and Nanping are the northern and western region.

### Inclusion and exclusion criteria

Inclusion Criteria: Patients with bacteriologically confirmed pulmonary tuberculosis who underwent rifampicin drug susceptibility testing (DST) with available results were included in the rifampicin resistance profile and epidemiological characteristics analysis. RR-TB patients who completed treatment and received outcome assessment were included in the treatment outcomes and influencing factors analysis.

Exclusion Criteria: Patients with non-tuberculous mycobacterial (NTM) infection confirmed by species identification or those lacking rifampicin DST results were excluded. RR-TB patients who did not complete treatment and consequently lacked outcome assessment were excluded from the treatment outcomes analysis.

### Drug resistance screening strategy and definitions

Drug resistance screening strategies in Fujian Province initially prioritized rifampicin resistance (RR) detection, with the Tuberculosis Information Management System (TBIMS) primarily documenting rifampicin susceptibility test results. In some regions, additional testing for isoniazid and fluoroquinolone (FQ) resistance was conducted, and these results were also recorded in the system. Since 2016, the scope of RR-TB screening has expanded progressively—from targeting only high-risk RR-TB populations to encompassing all bacteriologically confirmed TB cases, including new TB diagnoses. Furthermore, health authorities have advocated for the preferential use of rapid molecular diagnostic technologies for drug susceptibility testing (DST) to reduce diagnostic delays.

Bacteriologically confirmed TB was defined by a positive result on at least one of the following tests: smear microscopy, rapid molecular testing, or mycobacterial culture. New TB cases referred to patients with no history of anti-TB treatment or those who had received less than one month of therapy. High-risk groups for RR-TB included patients who remained bacteriologically positive after more than one month of anti-TB treatment, encompassing cases of treatment failure, relapse, retreatment after default, or other recurrent TB episodes. Treatment outcomes for RR-TB were categorized according to China’s Technical Specification for Tuberculosis Prevention and Control and included cure, treatment completion, treatment failure, death, and loss to follow-up. For analytical purposes, successful outcomes were defined as the composite of cured and treatment completed cases, while unsuccessful outcomes included death, treatment failure, and lost to follow-up.

### Drug susceptibility testing (DST) and quality control

Drug susceptibility testing was conducted in accordance with World Health Organization (WHO) guidelines using a comprehensive phenotypic and genotypic approach. Phenotypic DST was performed using the proportion method on Löwenstein-Jensen (L-J) medium with the following critical drug concentrations: rifampicin (1 μg /mL), isoniazid (0.2 μg/mL), and ofloxacin (4 μg /mL). Each testing batch included the *Mycobacterium tuberculosis* reference strain H37Rv as an internal quality control. For rapid detection of drug resistance, we employed molecular diagnostic platforms including: Xpert MTB/RIF Ultra® (*Cepheid, Sunnyvale, CA, USA*) for rifampicin resistance screening, MeltPro® TB assay (*Zeesan Biotech, Xiamen, China*) for comprehensive resistance profiling.

The laboratory examinations and quality control procedures were conducted in strict accordance with the relevant WHO guidelines. All participating laboratories successfully passed the drug susceptibility testing proficiency assessment administered by the National Tuberculosis Reference Laboratory in the preceding year, demonstrating their competency in strain identification and drug susceptibility testing.

### Data management and statistical analysis

Prior to analysis, the dataset underwent rigorous cleaning procedures to ensure data quality. For handling missing data, we will first verify it through the original dataset. If the data remains unavailable, the corresponding entry will be deleted. Duplicate case records are identified and resolved by merging or deletion. All statistical analyses were performed using SPSS version 24.0 (IBM Corp., Armonk, NY, USA). Descriptive statistics were calculated for all study variables. Continuous variables, such as age, were expressed as mean ± standard deviation (*SD*), while categorical variables, including gender, age group, occupation, household registration, and treatment history, were summarized as frequencies (*n*) with corresponding percentages (*%*).

Univariate analyses were conducted to evaluate potential associations between independent variables and RR-TB treatment outcomes. Variables demonstrating a significance level of *p* ≤ 0.20 in univariate analyses were subsequently included. A stepwise selection approach (with entry criteria *p* < 0.05 and exit criteria *p* > 0.10) was used in a multivariate logistic regression model to identify independent predictors. The regression analysis yielded both crude odds ratios (*ORs*) and adjusted odds ratios (*AORs*), accompanied by their respective 95% confidence intervals (*95% CIs*). A two-tailed *p*-value < 0.05 was considered statistically significant for all analyses. An *OR* > 1 indicates an increased likelihood of the outcome, while an *OR* < 1 suggests a decreased likelihood. Statistical significance was determined if the *95% CI* did not include 1 (equivalent to *p*-value < 0.05).

## Results

### Basic informations

From 2019 to 2024, a total of 37,329 bacteriologically confirmed TB patients were screened for rifampicin resistance in Fujian Province, including 31,744 new TB cases and 5,585 high-risk groups. There were 28,756 male TB patients, with a male-to-female ratio of 3.4:1. The mean (SD) age of patients was 53.38 (17.124) years, with the oldest patient being 97 years old and the youngest 8 months old. 84.8% of all patients used rapid DST, which was significantly exceeding that of traditional phenotypic DST (15.2%).

From 2019 to 2024, 37,329 culture-confirmed TB patients underwent rifampicin resistance screening in Fujian Province, comprising 31,744 (85.0%) new cases and 5,585 (15.0%) high-risk individuals. The population showed male predominance (28,756; 77.0%; male-to-female ratio 3.4:1) with a mean age of 53.38 years (SD 17.124; range 0.7–97 years). Rapid DST accounted for 84.8% of tests (31,654/37,329), significantly exceeding phenotypic DST utilization (15.2%; 5,675/37,329).

### Detection and characteristics of RR-TB

**Figure 1:**
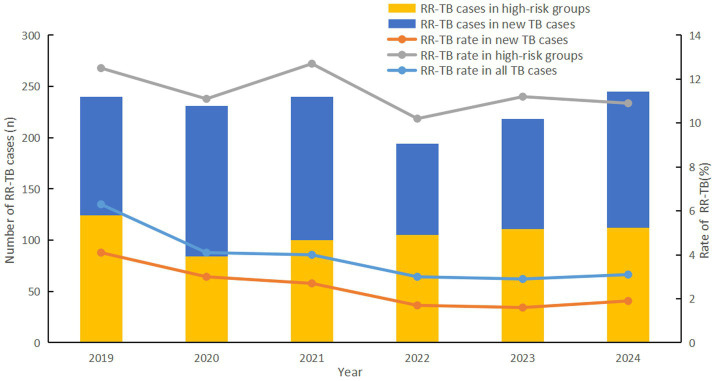
RR-TB found by year, Fujian province, Southeast China, 2019-2024.

A total of 1,368 RR-TB patients were detected, with an overall resistance rate of 3.7%. The rifampicin resistance (RR) rate showed a steady decline year by year, from 6.3% in 2019 to 3.1% in 2024 (*χ^2^* = 76.214, *p* < 0.001). Notably, this trend was largely attributable to a marked reduction in RR among new TB cases, where resistance rates dropped by 53.7%, from 4.1 to 1.9% (*χ^2^* = 60.966, *p* < 0.001). RR rate was not significantly reduced in high-risk groups (*χ^2^* = 1.538, *p* < 0.001), however it was significantly higher than in new TB cases (11.4% > 2.3%, *χ^2^* = 1109.551, *p* = 0.215) (Figure 1).

**Figure 2:**
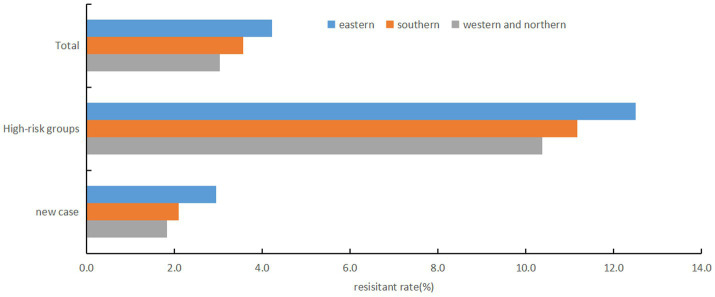
Regional disparities in rifampicin resistance rates, Fujian province, Southeast China, 2019-2024.

There were regional differences in RR rates (*χ^2^* = 16.987, *p* < 0.001), with the highest rate in eastern region (4.2%), followed by southern region (3.6%), and the lowest in northern and western region (3.0%) (Figure 2). No statistically significant difference was found in RR rates between male (2.3%) and female (2.4%) patients among new TB cases (*χ^2^* = 0.466, *p* = 0.495). However, in high-risk groups, females had a significantly higher RR-TB rate (14.3% vs. 10.8% in males, *χ^2^* = 9.213, *p* = 0.002). No significant differences were detected between Han Chinese (2.3% in new cases, 11.3% in high-risk groups) and other ethnic groups (2.2 and 16.5%, respectively) (*p* > 0.05). Among new TB cases, corporate and government employees had the highest RR rate (2.9%, *χ^2^* = 29.832, *p* < 0.001), whereas farmers and workers had the lowest (1.9%). In high-risk groups, unemployed and retired individuals showed the highest RR rate (16.4%, *χ^2^* = 14.768, *p* = 0.002). Non-local residents exhibited significantly higher RR rate compared to local residents in both new TB cases (4.1% vs. 1.6%) and high-risk groups (18.6% vs. 7.3%) (*χ^2^* = 174.646 and 161.689, respectively; *p* < 0.001). Younger individuals (≤44 years) had a higher RR rate than those ≥45 years in both new cases (2.8% vs. 2.1%) and high-risk groups (16.3% vs. 10.0%) (*χ^2^* = 14.068 and 37.767, respectively; *p* < 0.001) (Table 1).

**Table 1:** Prevalence of rifampicin resistance among tuberculosis (TB) patients by demographic and clinical characteristics in Fujian Province, Southeast China, 2019–2024.

Characteristics	New TB cases			High-risk groups			Total		
	RR-TB	*χ^2^*	*P*-value	RR-TB	*χ* ^2^	*P*-value	RR-TB	*χ* ^2^	*P*-value
Gender									
Female	184 (2.4)	0.466	0.495	133 (14.3)	9.213	0.002	317 (3.7)	0.034	0.853
Male	548 (2.3)			503 (10.8)			1,051 (3.7)		
Ethnic									
Han	720 (2.3)	0.049	0.825	622 (11.3)	2.21	0.137	1,342 (3.7)	0.301	0.583
Other	12 (2.2)			14 (16.5)			26 (4.1)		
Occupation									
Corporate or government employees	274 (2.9)	29.832	<0.001	201 (11.7)	14.768	0.002	475 (4.2)	25.789	<0.001
Farmers or workers	345 (1.9)			350 (10.5)			695 (3.2)		
Unemployed and retired	52 (2.8)			42 (16.4)			94 (4.5)		
Other job	61 (2.7)			43 (15.9)			104 (4.1)		
Household registration									
Local	363 (1.6)	174.646	<0.001	263 (7.3)	161.689	<0.001	626 (2.4)	161.66	<0.001
Non-local	369 (4.1)			373 (18.6)			742 (6.7)		
Age group									
~44	259 (2.8)	14.068	<0.001	200 (16.3)	37.767	<0.001	459 (4.4)	21.081	<0.001
45~	473 (2.1)			436 (10.0)			909 (3.4)		
Total	732 (2.3)			636 (11.4)			1,368 (3.7)		

Figures in brackets are rate (%).

Among the 1,368 rifampicin-resistant patients, 1,051 were male, with a male-to-female ratio of 3.1:1. The mean (*SD*) age of patients was 50.19 (15.548) years (range 10 - 91 years), and the majority of RR-TB patients (66.4%) were reported more than 45 years old. In terms of occupations, the majority of patients were farmers or workers (50.1%, *n* = 695), while 475 (34.7%) were unemployed or retired, 94 (6.9%) were employed in enterprises or government, and 104 (7.6%) were in other occupations. RR-TB patients with local household registration (54.2%, *n* = 742) were more than non-local (45.8%, *n* = 626). The Han ethnic group represented 98.1%. New cases accounted for 53.5% (*n* = 732), higher than high-risk groups (46.5%, *n* = 636).

### Other drug susceptibility testing

Among the patients, 20,439 (54.8%) underwent isoniazid susceptibility testing, with an overall resistance rate of 9.9%. The noteworthy finding is that both Isoniazid (71.9% > 6.3%, *χ^2^* = 5199.958, *p* < 0.001) and ofloxacin (29.8 > 2.7%, *χ^2^* = 902.975, *p* < 0.001)resistance rate in RR-TB patients were significantly higher than rifampicin-sensitive TB (RS-TB). Additionally, the isoniazid resistance rate of RR-TB in high-risk groups was higher than RR-TB in new cases (75% > 69.1%, *χ^2^* = 4.767, *p* = 0.029). And the ofloxacin resistance rate of RR-TB in high-risk groups was significantly higher than RR-TB in new cases (40.8% > 19.8%, *χ^2^* = 16.827, *p* < 0.001) (Table 2).

**Table 2:** Resistance patterns of isoniazid and ofloxacin among TB patients in Fujian Province, Southeast China, 2019–2024.

Registration category	Rifampicin DST result	Resistant to isoniazid			Resistant to ofloxacin		
		cases	*χ^2^*	*P*-value	cases	*χ^2^*	*P*-value
New TB cases	Sensitive	962 (6.0)	3059.053	<0.001	118 (1.5)	286.55	<0.001
	Resistance	419 (69.1)			33 (19.8)		
	Subtotal	1,381 (8.3)			151 (1.9)		
High-risk groups	Sensitive	246 (7.5)	1481.861	<0.001	53 (3.1)	343.306	<0.001
	Resistance	404 (75.0)			62 (40.8)		
	Subtotal	650 (17.1)			115 (6.2)		
Total	Sensitive	1,208 (6.3)	5199.958	<0.001	171 (1.8)	902.975	<0.001
	Resistance	823 (71.9)			95 (29.8)		
	Subtotal	2031 (9.9)			266 (2.7)		

Figures in brackets are rate (%).

### Treatment outcomes and influencing factors analysis

A total of 1,056 RR-TB patients were included in treatment, accounting for 77.2%. Among them, 720 RR-TB patients had completed treatment outcome assessment, 422 were successfully treated, with a successful treatment rate of 58.6%, including 220 treatment completed (30.6%) and 202 cured (28.1%). The lower treatment success rate was due to the higher proportion of loss of follow-up (224, 31.1%) and deaths (67, 9.3%) (Table 3).

**Table 3:** Treatment outcomes of rifampicin-resistant tuberculosis (RR-TB) patients in Fujian Province, Southeast China, 2019–2024.

Outcomes	Cases	Rate (%)
Successful	422	58.6
Treatment completed	220	30.6
Cured	202	28.1
Unsuccessful	298	41.4
Lost to follow-up	224	31.1
Death	67	9.3
Failure	7	1.0

Univariate analysis found that there was no significant difference in treatment success rates between RR-TB patients with different ethnic or different household registration.

Univariate analysis revealed no significant differences in treatment success rates among RR-TB patients when stratified by ethnicity or household registration status. Consequently, these two variables were excluded from subsequent multivariate logistic regression analysis, while all other variables were included.

The multivariate logistic regression model showed significant predictive power (*χ^2^* = 61.166, *df* = 6, *p* < 0.001). While demonstrating good fit (Hosmer-Lemeshow *χ^2^* = 1.268, *p* = 0.989), the model explained only 11% of variance (Nagelkerke *R^2^* = 0.110), suggesting limited explanatory power of the current predictors. The regression analysis identified gender, age, registration category, and occupation as independent risk factors for unsuccessful treatment in RR-TB patients. Male patients had 1.673 -fold higher risk of unsuccessful treatment compared to female patients (*AOR* = 1.673, 95% *CI* = 1.111–2.520, *p* = 0.014). Patients aged ≥45 years had 2.271-fold increased risk of unsuccessful treatment compared to those <45 years (*AOR* = 2.271, *95% CI* = 1.584–3.255, *p* < 0.001). Patients in high-risk groups had 1.420-fold higher risk of unsuccessful treatment compared to new TB cases (*AOR* = 1.420, *95% CI* = 1.042–1.935, *p* = 0.026). Patients with occupations as farmers or workers had 2.165-fold greater risk of unsuccessful treatment compared to those with occupations as unemployed or retired (*AOR* = 2.165, *95% CI* = 1.100–4.259, *p* = 0.025) (Table 4).

**Table 4:** Univariate and multivariate logistic regression analysis of risk factors associated with unsuccessful treatment outcomes in rifampicin-resistant tuberculosis (RR-TB) patients, Fujian Province, Southeast China, 2019–2024.

Variables	Outcomes			Univariate analysis		Multivariate analysis	
	Successful	Unsuccessful	Successful rate (%)	Crude OR (95% CI)	*P*-value	Adjust OR (95% CI)	*P*-value
Gender							
Female	121	44	73.3	1		1	
Male	301	254	54.2	2.321 (1.582–3.405)	<0.001	1.673 (1.111–2.520)	0.014
Ethnic							
Han	417	292	58.5	1		--	--
Other	5	6	45.5	1.714 (0.518–5.668)	0.377	--	--
Age group							
~44	184	63	74.5	1		1	
45~	238	235	50.3	2.884 (2.056–4.044)	<0.001	2.271 (1.584–3.255)	<0.001
Occupation							
Corporate or government employees	46	13	78.0	1		1	
Farmers or workers	188	176	51.6	3.313 (1.731–6.339)	<0.001	2.165 (1.100–4.259)	0.025
Unemployed or retired	149	92	61.8	2.185 (1.120–4.262)	0.022	1.690 (0.847–3.374)	0.136
Other job	39	17	69.6	1.542 (0.667–3.568)	0.311	1.270 (0.535–3.014)	0.588
Household registration							
Local	177	125	58.6	1		--	--
Non-local	245	173	58.6	1.000 (0.740–1.350)	0.999	--	--
Registration category							
New TB cases	234	138	62.9	1		1	
High-risk groups	188	160	54.0	1.443 (1.071–1.944)	0.016	1.420 (1.042–1.935)	0.026

## Discussion

In 2019, the Fujian Provincial Government launched the “Action Plan to End Tuberculosis (2019–2022)” and set ambitious targets for rifampicin resistance testing. The new goal was to screen at least 90% of bacteriologically confirmed TB cases for rifampicin resistance by 2022. Based on province-wide drug resistance screening data, this study revealed that the overall rifampicin resistance rate among culture-positive pulmonary TB cases in Fujian Province was 3.7%, showing a steady year-on-year decline. The resistance rates in both new TB cases and high-risk groups were lower than those reported nationally and in other provinces (9–13). The decline was primarily observed in new TB cases, while the resistance rate in high-risk groups remained relatively stable, indicating some success in curbing the transmission of RR-TB. Although the resistance rate in high-risk groups was significantly higher than in new patients, the absolute number of RR-TB cases was greater among new TB cases due to their substantially larger case population. Therefore, our current strategy emphasizes equal attention to both populations in prevention and screening efforts.

Geographical analysis revealed regional variations in rifampicin resistance rates, with higher rates in eastern Fujian compared to southern, northern and western regions. This disparity may be attributed to economic and demographic factors, as eastern Fujian is more economically developed, with greater population mobility and density, which increase the risk of RR-TB transmission. Additionally, studies suggest that the concentration of medical resources in economically developed areas may lead to cross-regional healthcare-seeking behavior, potentially facilitating the spread of RR-TB (14, 15).

The majority of RR-TB patients in Fujian were male, aged 45 years or older, farmers or workers, and non-local residents. These demographic characteristics may be associated with lifestyle factors (e.g., smoking, alcohol use), broader social networks, and the higher prevalence of comorbidities (e.g., diabetes, chronic obstructive pulmonary disease) in older adults, all of which may increase the risk of infection and transmission of drug-resistant strains (16, 17). However, the higher RR-TB rate among females, unemployed and retired individuals or younger individuals (<45 years) also warrant close attention. Given these findings, targeted interventions, including enhanced screening strategies, should be implemented to facilitate early detection and control of RR-TB in these groups.

The study found that RR-TB patients exhibited higher rates of resistance to isoniazid and ofloxacin compared to RS-TB patients. This pattern may reflect the longer treatment histories of RR-TB patients, increasing their exposure to various drugs and the likelihood of developing resistance (18). The isoniazid resistance rate among RR-TB patients reached 71.9%, consistent with global reports indicating that 78% of new RR-TB cases are MDR-TB (19). The high isoniazid co-resistance rate in RR-TB patients likely reflects acquired resistance due to prior inadequate treatment. Since isoniazid and rifampicin are core first-line anti-TB drugs frequently administered in combination, their concurrent use exerts substantial selective pressure for the emergence of dual resistance. This risk is particularly heightened in cases of irregular or incomplete treatment, often resulting in treatment failure and the development of multidrug-resistant tuberculosis (MDR-TB) (20, 21). Supporting this, in high-risk groups showed higher odds of co-resistance than new cases, underscoring the role of treatment failures in driving MDR development. As isoniazid and rifampicin are core anti-TB drugs often used together throughout treatment, there is significant selective pressure for dual resistance, particularly in cases of irregular treatment (22). Notably, ofloxacin resistance was also prevalent among RR-TB patients. As a key fluoroquinolone, ofloxacin exhibits incomplete cross-resistance with other fluoroquinolones in RR-TB treatment (23). Without fluoroquinolone susceptibility testing, the use of fluoroquinolone-containing regimens may compromise treatment success rates and potentially lead to additional drug resistance. Therefore, in light of the high isoniazid and ofloxacin resistance rates in Fujian, it is essential to modify existing drug resistance screening protocols to include systematic testing for both agents in RR-TB cases. Moreover, individualized RR-TB treatment regimens should be optimized based on detailed susceptibility profiles for fluoroquinolones and isoniazid to ensure therapeutic efficacy.

The treatment rate for RR-TB patients in Fujian Province was only 77%, below the national average of 79.38% in 2021 (24), and the treatment success rate was similarly low at 58.6%. Compared to several eastern provinces in China, Fujian Province demonstrates a relatively lower success rate in the treatment of rifampicin-resistant tuberculosis (RR-TB). This disparity may be attributed to differences in healthcare infrastructure, diagnostic capacity, or treatment adherence protocols (15, 25, 26). Loss to follow-up was the primary reason for unsuccessful treatment in Fujian Province. Previous researches have demonstrated that loss to follow-up among RR-TB patients is influenced by a range of independent risk factors. These include structural barriers (e.g., distance from treatment centers, rural residence), patient demographics (older age, male sex), and clinical factors (extrapulmonary TB, HIV coinfection, multidrug resistance, and delayed sputum culture conversion). Additionally, psychosocial challenges (negative treatment attitudes, limited social support), health system-related issues (dissatisfaction with care), socioeconomic disadvantage, and treatment-related adverse events further contribute to loss to follow-up (27, 28). These individuals represent significant sources of RR-TB transmission, necessitating further research and Addressing these multifaceted determinants is critical to improving RR-TB treatment adherence and outcomes.

Univariate and multivariate logistic regression analyses identified gender, age, registration category, and occupation as independent factors influencing treatment outcomes in rifampicin-resistant patients. Male, age ≥45 years, high-risk group status, and migrant worker occupation were identified as risk factors for unsuccessful treatment. Previous studies suggest that declining immunity and comorbidities in older adults complicate the clinical presentation and management of RR-TB. Gender significantly influences treatment outcomes, with male patients more likely to have unhealthy habits (e.g., smoking, alcohol use) and delayed healthcare-seeking behavior. Farmers and workers face greater economic pressures and mobility, increasing the risk of treatment interruption and loss to follow-up. High-risk groups, particularly those with multiple previous TB treatments, often harbor more complex drug resistance patterns, all of which contribute to poor treatment outcomes (29–35). Targeted interventions for these populations should be implemented within existing TB control programs to improve treatment adherence and reduce the risk of unfavorable outcomes. The recommended interventions include implement comprehensive geriatric assessments with integrated comorbidity management and nutritional supplementation programs for older adult patients, develop gender-specific behavioral interventions including smoking cessation programs and male-focused health education initiatives to improve healthcare utilization, provide economic subsidies and optimize trans-regional treatment management for Farmers and workers, introduce enhanced treatment monitoring protocols with more frequent follow-ups and expanded drug susceptibility testing for high-risk groups.

Several limitations should be considered when interpreting our findings. First, potential selection bias may have been introduced through loss to follow-up, which could impact the generalizability of our results. Notably, if patients who were lost to follow-up sought treatment elsewhere and achieved favorable outcomes, our estimates of treatment success rates may be conservative. Second, constrained by the nature of our data sources, this study had limited capacity to examine potentially important confounding variables, particularly regarding comorbidities such as diabetes and HIV status. Future studies would benefit from prospective designs that incorporate more comprehensive clinical data collection, including detailed comorbidity profiles, to facilitate more robust analyses of these potential confounders.

## Conclusion

The rifampicin resistance (RR) rate demonstrated a consistent year-on-year decline, falling from 6.3% in 2019 to 3.1% in 2024 in Fujian Province—a trend driven largely by a 53.7% reduction in RR among new TB cases (4.1 to 1.9%). The high prevalence of isoniazid and ofloxacin resistance among RR-TB cases necessitates revising current drug resistance screening to include systematic testing for both agents in RR-TB cases. The low treatment and success rates for RR-TB patients highlight the need for improved strategies to ensure comprehensive treatment coverage and enhance treatment outcomes. Special attention should be given to male patients, those aged ≥45 years, high-risk groups and farmers or workers, who face higher risks of poor treatment outcomes. Targeted interventions such as comorbidity management, nutritional support and economic subsidies should be implemented for these patient populations to improve treatment adherence and reduce unfavorable treatment outcomes.

## Data Availability

The original contributions presented in the study are included in the article/supplementary material, further inquiries can be directed to the corresponding author.
